# Ammonia Sensing Behaviors of TiO_2_-PANI/PA6 Composite Nanofibers

**DOI:** 10.3390/s121217046

**Published:** 2012-12-12

**Authors:** Qingqing Wang, Xianjun Dong, Zengyuan Pang, Yuanzhi Du, Xin Xia, Qufu Wei, Fenglin Huang

**Affiliations:** Key Laboratory of Eco-Textiles, Ministry of Education, Jiangnan University, Wuxi 214122, Jiangsu, China; E-Mails: wqq888217@126.com (Q.W.); crivia1988@hotmail.com (X.D.); pangzengyuan1212@163.com (Z.P.); dyzddd@163.com (Y.D.); xjxiaxin@163.com (X.X.); windhuang325@163.com (F.H.)

**Keywords:** electrospinning, sputtering, nanofiber, sensor

## Abstract

Titanium dioxide-polyaniline/polyamide 6 (TiO_2_-PANI/PA6) composite nanofibers were prepared by *in situ* polymerization of aniline in the presence of PA6 nanofibers and a sputtering-deposition process with a high purity titanium sputtering target. TiO_2_-PANI/PA6 composite nanofibers and PANI/PA6 composite nanofibers were fabricated for ammonia gas sensing. The ammonia sensing behaviors of the sensors were examined at room temperature. All the results indicated that the ammonia sensing property of TiO_2_-PANI/PA6 composite nanofibers was superior to that of PANI/PA6 composite nanofibers. TiO_2_-PANI/PA6 composite nanofibers had good selectivity to ammonia. It was also found that the content of TiO_2_ had a great influence on both the morphology and the sensing property of TiO_2_-PANI/PA6 composite nanofibers.

## 1. Introduction

Ammonia is a toxic gas with very penetrating odor. High concentrations of ammonia constitutea threat to human health. Exposure to high ammonia concentrations of 1,000 ppm or more can cause pulmonaryoedema and accumulation of fluid in the lungs, leading to difficulty with breathing and tightness in the chest. Today, most of the ammonia in our atmosphere is emitted directly or indirectly by human activity. The majority of all man-made ammonia is used for the production of fertilizers and for use in refrigeration systems [1]. Because the chemical industry, fertilizer factories and refrigeration systems make use of almost pure ammonia, a leak in the system, especially in ammonia production plants where ammonia is produced, can result in life-threatening situations.

Conducting polymers such as polypyrrole, polyaniline, polythiophene and their derivatives are being explored as promising materials for microsensors, because of their good ability to form chemical sensors either as a sensing element or as matrices to immobilize specific reagents. Among these conducting polymers, polyaniline nanomaterials are the most extensively studied because of their greater surface area that allows fast diffusion of gas molecules into the structure [2], and they have been successfully demonstrated as efficient gas sensors for monitoring airborne organic and inorganic components such as alcohol vapor [3,4], methanol [5], hydrogen [6,7], aromatic organic compounds (AOCs) [8], chloroform vapor [9,10], NO_2_[11], and especially ammonia [12–18]. Three major kinds of PANI-based ammonia sensing composite materials are described in the literature, including PANI-polymer composite [19,20], PANI-CNTs (or PANI-MWCNTs) composite [21,22] and PANI-metal dioxide composites (such as PANI-SnO_2_[23], PANI-In_2_O_3_[23], PANI-ZnO [24] and PANI-TiO_2_[25,26]). Recently, more attention has been given to composite materials of PANI and metal dioxide, and ammonia sensing composites based on PANI and TiO_2_.

In this work, PA6 nanofibers obtained by an electrospinning technique were first used as template to fabricate PANI/PA6 composite nanofibers by *in situ* polymerization. Then, TiO_2_-PANI/PA6 composite nanofibers were prepared by depositing TiO_2_ onto the PANI/PA6 substrate via RF magnetron sputtering. The TiO_2_-PANI/PA6 composite nanofibers were finally fabricated into sensing devices for sensing application.

## 2. Experimental Section

### 2.1. Materials

Aniline, formic acid (FA), ammonium persulfate (APS), ammonia and sulfuric acid (H_2_SO_4_) were purchased from Sinopharm Chemical Reagent Co. Ltd. (Beijing, China). Polyamide 6 (PA6, Mw = 21,000 g/mol) was obtained from ZIG ZHENG Industrial Co. Ltd (Taibei, Taiwan). All chemicals and reagents were used as received, except for aniline, which was distilled before use.

### 2.2. Fabrication of PANI/PA6 Composite Nanofibers

**Figure 1.**
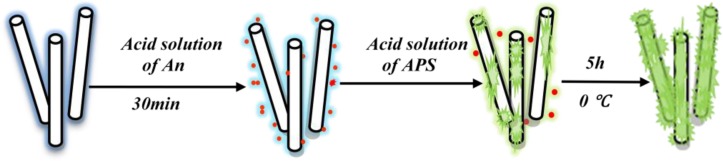
Fabrication of PANI/PA6 composite nanofibers.

PANI/PA6 composite nanofibers were prepared by electrospinning and *in situ* polymerization. PA6 nanofibers were firstly prepared by electrospinning PA6/FA solutions of 20% PA6 concentration. Then, aniline and APS were dissolved separately in aqueous solutions, and the H^+^ concentrations of the aqueous solutions were adjusted by H_2_SO_4_ to 1.5 mol/L. The mole ratio of aniline to APS was 1:1. The electrospun PA6 nanofibers, were then immersed into the aniline/H_2_SO_4_ solution for 30 min. Successive polymerization was finally initiated by dropping the acid aqueous solution of APS into the above diffusion bath. PANI was synthesized on the surface of PA6 nanofibers and doped with H_2_SO_4_ at 0–5 °C for 5 h. After the reaction, the samples were taken out, washed with deionized water, and dried in vacuum at 65 °C for 12 h. The steps for synthesis of PANI/PA6 composite nanofibers are illustrated in Figure 1.

### 2.3. Fabrication of TiO_2_-PANI/PA6 Composite Nanofibers

TiO_2_-PANI/PA6 composite nanofibers were obtained by depositing TiO_2_ onto PANI/PA6 substrate at room temperature for different times via RF magnetron sputtering. High purity titanium discs (99.99%) of 50 mm diameter was used as sputtering targets. High purity argon (99.999%) and oxygen (99.999%) were used as the sputtering and reactive gases, respectively. A diffusion pump was used to get the desired 9.8 × 10^−4^ Pa base pressure. The argon and oxygen flow rates were controlled separately by mass flow meters. The distance between target and substrates was kept at 60 mm. Before each sputtering-deposition step, the target was pre-sputtered in argon for 10 min to clean the target surface. The sputtering conditions are listed in Table 1.

### 2.4. Characterization

The structure and surface morphology of PANI/PA6 and TiO_2_-PANI/PA6 composite nanofibers were observed with a field emission scanning electron microscope (FESEM, S-4800, Hitachi, Tokyo, Japan) with a golden coating. Fourier transform infrared (FTIR) spectrum of all the samples was obtained with a resolution of 4 cm^−1^ in the range of 400–4,000 cm^−1^ by using a NEXUS 470 spectrometer (Nicolet, Madison, WI, USA).

### 2.5. Gas Sensing Test

**Figure 2.**
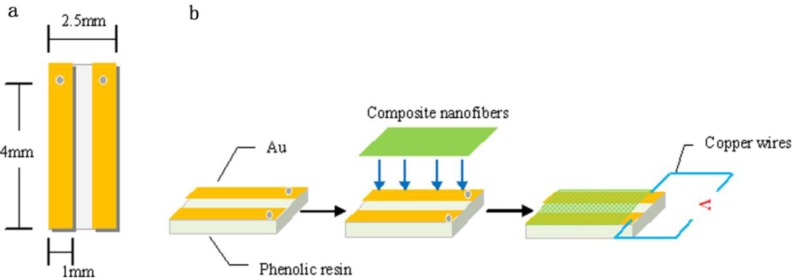
Schematic illustration of (**a**) home-made Au electrode and (**b**) sensing electrode.

A sensing system for ammonia sensing test was fabricated by the following processes. A home-made Au electrode with a gap of 0.5 mm between two Au stripes was firstly prepared by depositing Au on phenolic resin, and then PANI/PA6 and TiO_2_-PANI/PA6 composite nanofibers were pasted onto the open area between the two electrodes as shown in Figure 2.

**Figure 3.**
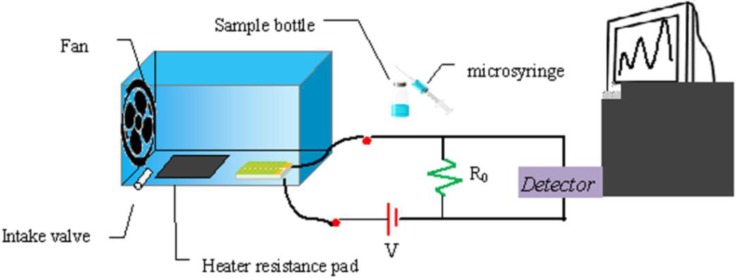
Schematic illustration of lab-made sensing system.

To test the ammonia sensitivity, the sensing electrode was placed in a lab-made sensing system as shown in Figure 3. The sensing set-up consisted of an airtight test chamber with 4,500 mL volume, a heater pad and a fan. Two minutes after the system reached a steady-state, a certain amount of ammonia was injected with a microsyringe through the intake valve, and with the help of a heater pad, the ammonia was heated to evaporation. The resistance changes of PANI/PA6 and TiO_2_-PANI/PA6 composite nanofibers sensors were monitored and recorded automatically by an Agilent electrometer and a computer. A constant voltage of 5.0 V was used as the DC power supply. All the tests were conducted at room temperature (25 ± 1 °C) with a relative humidity of 65 ± 1%.

During the measurements, the actual ammonia volumes injected were 0.67, 1.35, 2.02, 2.69 and 3.37 μL, corresponding to the ammonia vapor with the concentration of 50, 100, 150, 200 and 250 ppm, respectively. After the ammonia was introduced to the chamber, the resistance of the sensors was recorded for 250 s, then the test chamber was flushed with dry air consecutively for another 250 s to make sure that a relatively steady state had achieved before next cyclic test. The sensitivity is defined as (R_i_ – R_0_)/R_0_, where R_i_ and R_0_ are the resistance of sensors in ammonia and in air, respectively. The response and recovery time are defined as the time of 90% total resistance change. Each result was the average value of fivetimed tests.

## 3. Results and Discussion

### 3.1. Surface Morphology

**Figure 4.**
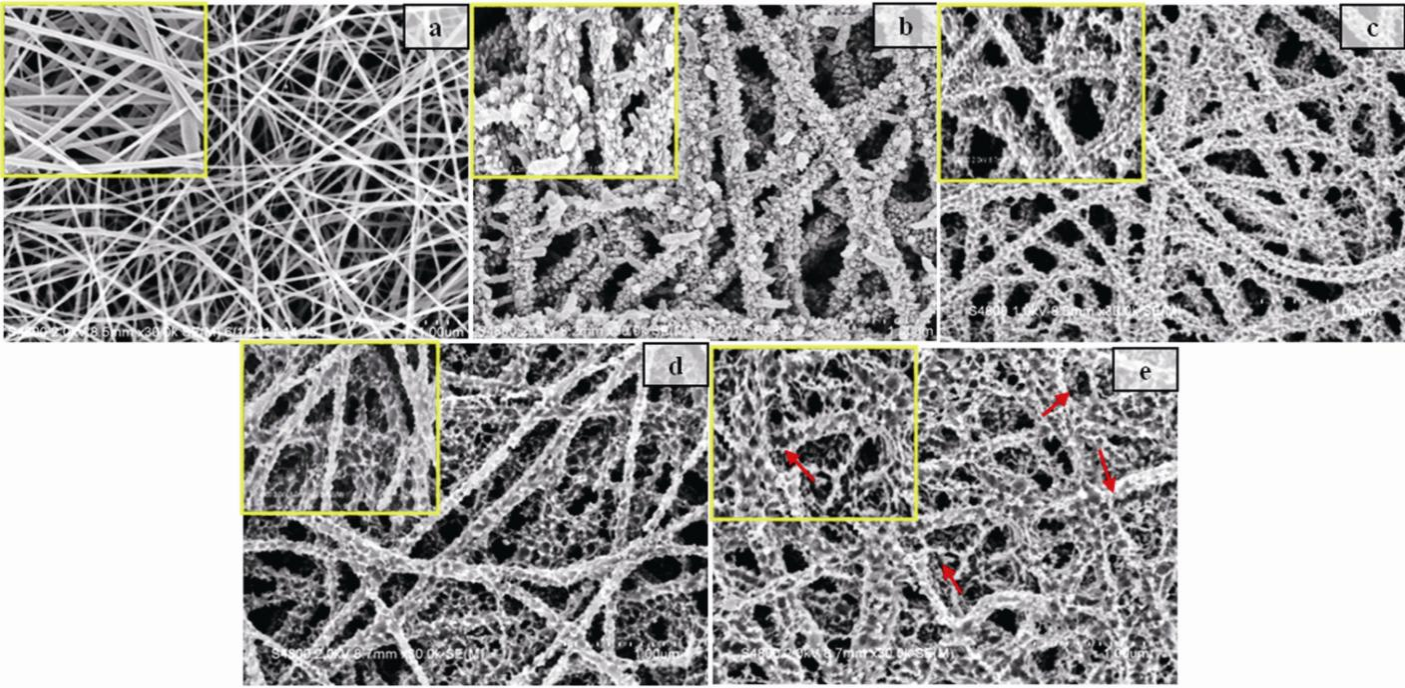
SEM image of (**a**) PA6 nanofibers; (**b**) PANI/PA6 nanofibers; (**c**) PANI/PA6 nanofibers sputtered for 30 min; (**d**) TiO_2_-PANI/PA6 nanofibers sputtered for 60 min, (**e**) TiO_2_-PANI/PA6nanofibers sputtered for 90 min.

It is well known that structure and morphology can have a significant effect on the sensing properties of materials. The SEM images of PA6 nanofibers, PANI/PA6 composite nanofibers and TiO_2_-PANI/PA6 composite nanofibers sputtered for different times are shown in Figure 4. It can be seen that the surface morphology of PA6nanofibersappeared smooth, while PANI/PA6 composite nanofibers had a rough surface and more uniform diameter because of the PANI coating, as indicated in Figure 4(a,b). The SEM images clearly revealed that the TiO_2_-PANI/PA6 composite nanofibers had very rough surface with porous structures, as presented in Figure 4(c–e). This could be attributed to the impact of high-energy particles during sputtering deposition of TiO_2_. It is obvious that such porous structure exhibited higher specific surface area than PANI/PA6, which facilitated the diffusion of ammonia vapor in sensing materials. However, TiO_2_-PANI/PA6 with 90 min deposition time showed a distorted surface structure as the excessive sputtering time could damage the integrity of the PANI coating.

### 3.2. FTIR Analysis

**Figure 5.**
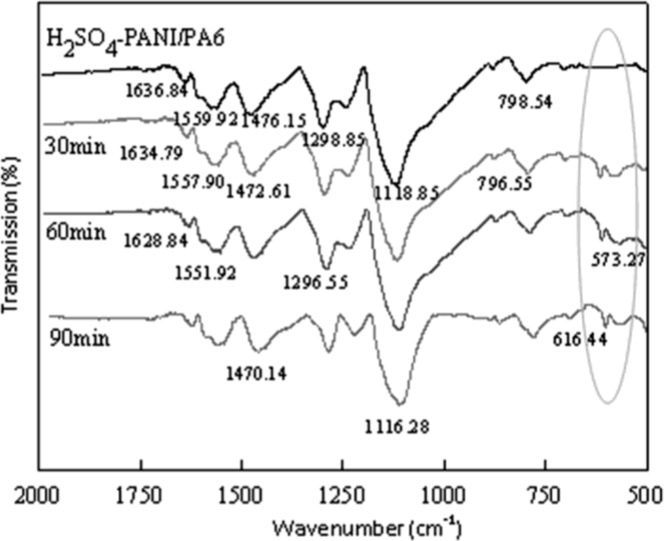
FTIR spectra of PANI/PA6 and TiO_2_-PANI/PA6 sputtered for 30 min, 60 min and 90 min, respectively.

The FTIR spectra of PANI/PA6 and TiO_2_-PANI/PA6 composite nanofibers are shown in Figure 5. For the PANI/PA6 composite nanofibers, the peaks around 1,476.15 cm^−1^ and 1,559.92 cm^−1^ were assigned to C=C stretching vibrations of the benzenoid and quinoid rings, respectively. The peaks at 1,298.85 cm^−1^ and 798.54 cm^−1^ resulted from the C–N stretching vibration of the secondary aromatic amine and the C–H bending vibration, respectively. The characteristic peak of Q=NH^+^–B was also observed at around 1,118.85 cm^−1^. All these peaks were identical to those of PANI. On the other hand, the C=O stretching vibration peak of amide in PA6 was also observed at 1,636.84 cm^−1^, while the amide N–H stretching vibration peak overlapped with the C=C stretching peak of the PANI quinoid rings, thus the peak at 1,559.92 cm^−1^ was shown to be broader. All the characteristic peaks of PANI/PA6 could be observed in the spectrum of TiO_2_-PANI/PA6 composite nanofibers, and the characteristic bands around 616.44 cm^−1^ and 573.27 cm^−1^ was attributed to Ti–O bending vibration of TiO_2_. It also can be observed that incorporation of TiO_2_ nanoparticles leads peaks of PANI/PA6 to shift slightly to lower wave number, indicating that some interaction existed between PANI and TiO_2_.

### 3.3. Gas Response Behavior of Sensors

#### 3.3.1. Effect of Sputtering Time

**Figure 6.**
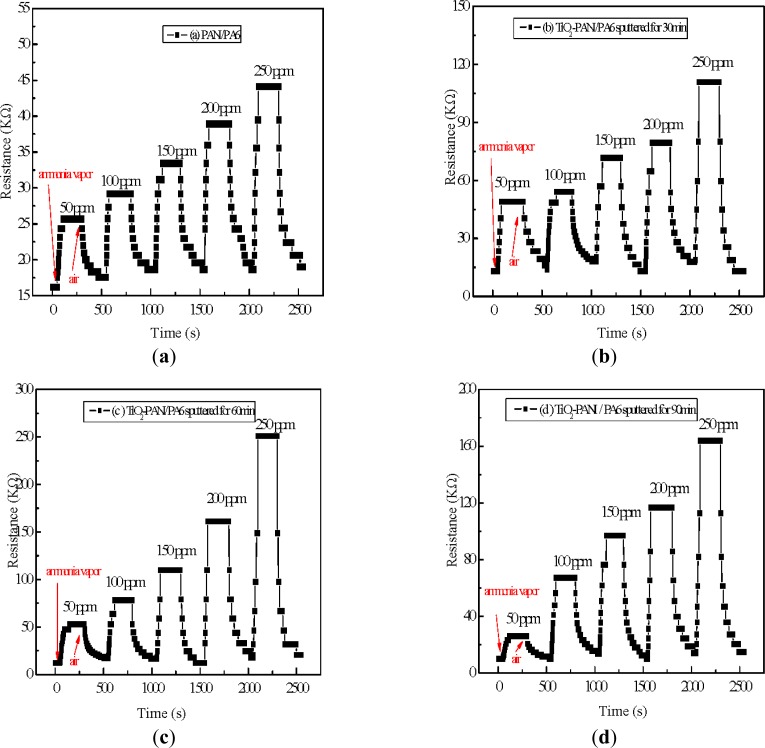
Dynamic response and recovery of (**a**) PANI/PA6; (**b**) TiO_2_-PANI/PA6 sputtered for 30 min; (**c**) TiO_2_-PANI/PA6 sputtered for 60 min and (**d**) TiO_2_-PANI/PA6 sputtered for 90 min to ammonia vapor of different concentrations.

**Figure 7.**
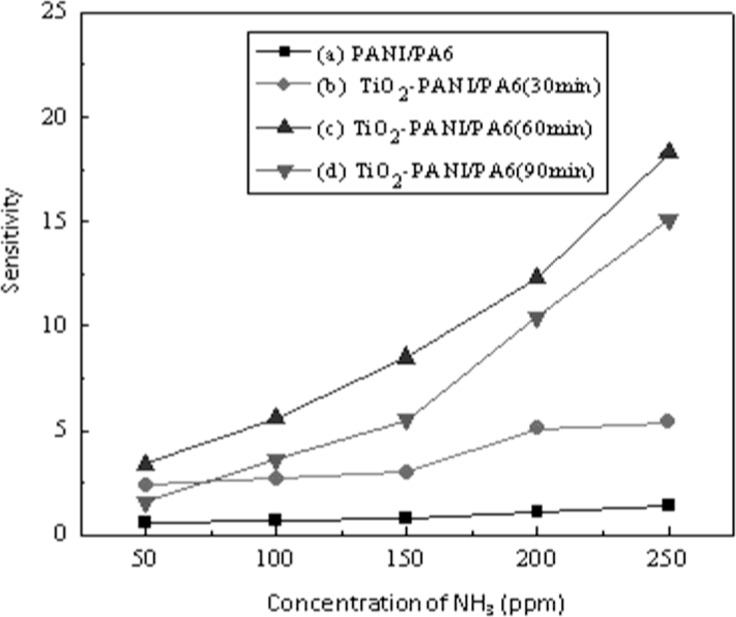
Sensitivity of (a) PANI/PA6; (b) TiO_2_-PANI/PA6 sputtered for 30 min; (c) TiO_2_-PANI/PA6 sputtered for 60 min and (d) TiO_2_-PANI/PA6 sputtered for 90 min to ammonia vapor of different concentrations.

To investigate the effect of TiO_2_nanopaticles on the ammonia sensing properties, ammonia sensing comparison tests were carried out with PANI/PA6 and TiO_2_-PANI/PA6 sputtered for 30, 60 and 90 min. Figure 6 shows the dynamic response-recovery of all samples to 50, 100, 150, 200 and 250 ppm ammonia vapor. It can be seen that resistance of all samples increased dramatically when exposed to ammonia vapor and decreased gradually when dry air was introduced. Compared to PANI/PA6 nanofibers, all TiO_2_-PANI/PA6 composite nanofibers showed better ammonia sensitivity, as revealed in Figure 7. It is obvious that the sensitivity of ammonia sensing material improved greatly after TiO_2_ deposition. It is also found that TiO_2_-PANI/PA6 sputtered for 60 min performed best among the samples prepared. Taking 250 ppm ammonia vapor for instance, the sensitivity of PANI/PA6 composite nanofibers was only 1.4, but the sensitivity of TiO_2_-PANI/PA6composite nanofibers sputtered for 30 min increased to 5.2, and the sensitivity of TiO_2_-PANI/PA6 sputtered for 60 min was as high as 18.3. However, when the sputtering time was extended to 90 min, the sensitivity of TiO_2_-PANI/PA6 composite nanofibers was 15.1 with a small decline.

**Figure 8.**
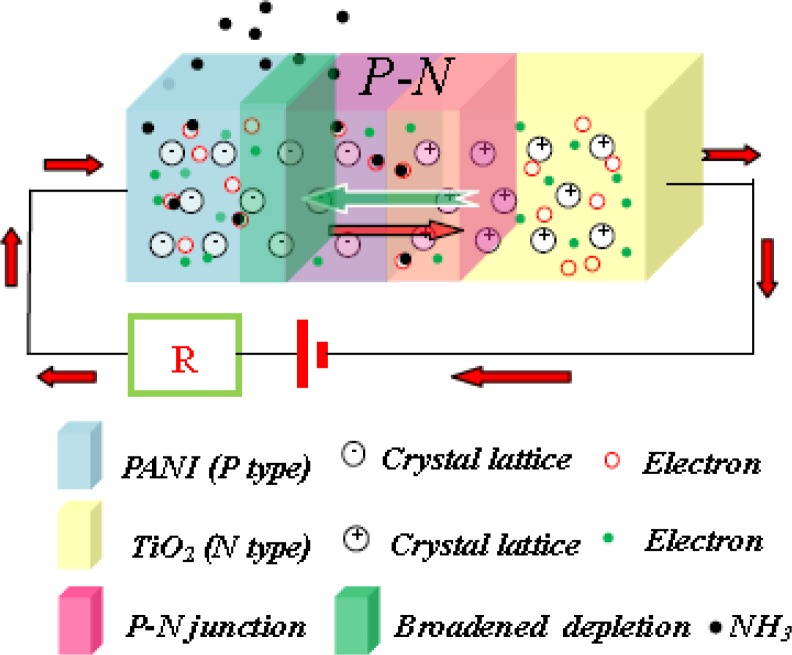
The effect of NH_3_ on the depletion layer of TiO_2_-PANI P–N junction.

TiO_2_ nanoparticles were deposited randomly on the PANI/PA6 substrate, which contributed to the good contact between TiO_2_ and PANI. Sensing properties of PANI/PA6 were due to the reversible chemisorptions of PANI [27,28], while the ammonia sensing behavior of TiO_2_-PANI/PA6 composite nanofibers was the joint function of PANI and P-N junction formed between p-type PANI and n-type TiO_2_. When exposed to the ammonia vapor, PANI was deprotonated by ammonia, which would increase the resistance of PANI and broaden the depletion layer thickness of P–N junction, as shown in Figure 8. The change of depletion layer thickness would increase the resistance of P–N junction. Therefore, the resistance changes in both PANI and P–N junction play a key role in controlling the current through the P–N composite sensor. In addition, because the absorption of ammonia would not only change the conductivity of PANI particles but also the resistance of P–N junction, the increase of TiO_2_ content in the nanofibers could result in an increase in the sensitivity of the nanofiber sensors at low ranges of TiO_2_ content. However, too high a content of TiO_2_ resulting from overlong sputtering times would damage the continuous phase of PANI layer, as revealed in Figure 4(e).

#### 3.3.2. Sensing Cyclability of Composite Nanofibers

**Figure 9.**
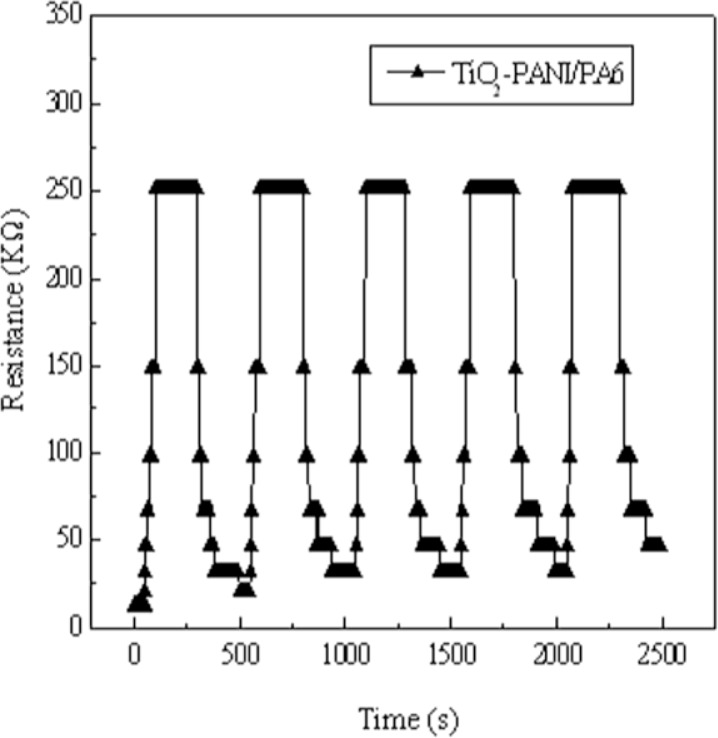
Cyclability of TiO_2_-PANI/PA6 to ammonia vapor of 250 ppm.

The reliability of sensing materials also depends on their repeated use. Figure 9 depicts the response of TiO_2_-PANI/PA6 composite nanofibers sputtered for 60 min to successive exposures to 250 ppm ammonia vapor. The resistance remained constant after repeat uses, indicating the good reproducibility of the material.

#### 3.3.3. Selectivity of TiO_2_-PANI/PA6 Composite Nanofibers

**Figure 10.**
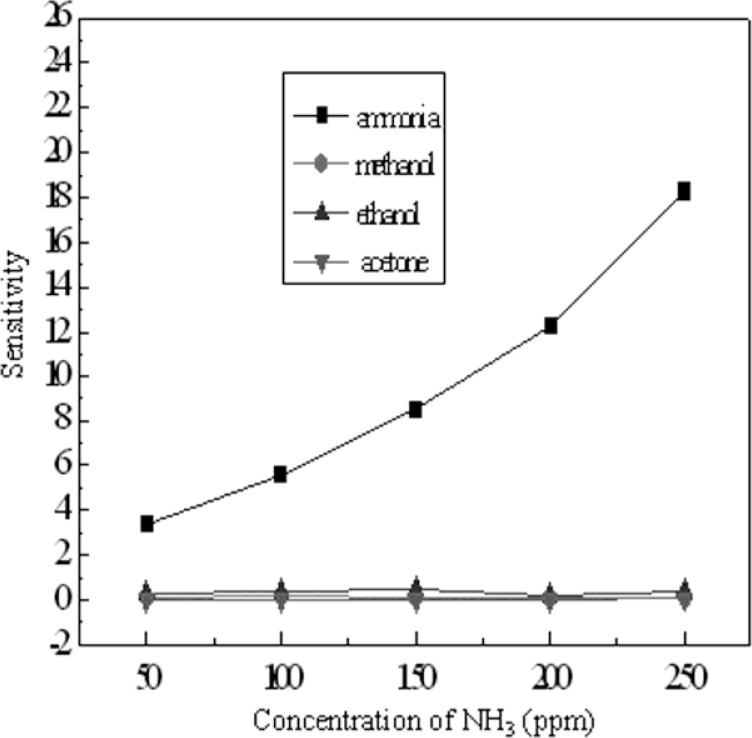
Selectivity of TiO_2_-PANI/PA6 to ammonia, methanol, ethanol and acetone.

Methanol, ethanol and acetone are common volatile liquids, whose vapor could show cross-sensitivity in the detection system. Therefore, in this work, the response and recovery experiments of TiO_2_-PANI/PA6 composite nanofibers to methanol, ethanol and acetone vapor in the range of 50–250 ppm were also conducted under the same conditions as the ammonia sensing experiments explained before. Figure 10 presents the sensitivity of TiO_2_-PANI/PA6 composite nanofibers sputtered for 60 min to ammonia, methanol, and ethanol and acetone vapors. It is very obvious that the sensitivities of the sensor to methanol, ethanol and acetone vapors were much lower than those to ammonia, which indicates the selective sensing behavior of the TiO_2_-PANI/PA6 composite nanofibers. The TiO_2_-PANI/PA6 composite nanofibers thus showed excellent selectivity to ammonia vapor.

## 4. Conclusions

From the above mentioned studies, it has been concluded that TiO_2_-PANI/PA6 composite nanofibers were successfully fabricated via the combination of *in situ* chemical polymerization and sputtering, which was a new, easy-to-handle and inexpensive technique. P-N junctions formed between PANI and TiO_2_ played a key role in the sensing behavior of TiO_2_-PANI/PA6 composite nanofibers to ammonia, which lead to a better sensitivity to ammonia such as higher response sensitivity, better response and recovery, compared to PANI/PA6 composite nanofibers. It clearly appeared that the content of TiO_2_ component controlled by sputtering-deposition time influenced the morphology and sensing property of TiO_2_-PANI/PA6 composite nanofibers. The gas-sensing properties of TiO_2_-PANI/PA6 composite nanofibers to ammonia, methanol, and ethanol and acetone vapor indicated that TiO_2_-PANI/PA6 composite nanofibers had excellent selectivity for ammonia detection, but would not applicable for the fabrication of methanol, ethanol and acetone vapor sensors. Further work will be devoted to improving the stability of the TiO_2_-PANI/PA6 composite nanofibers sensor.

## Figures and Tables

**Table 1.** Sputtering conditions.

**Deposition time (*min*)**	**Sputtering power (*W*)**	**Total pressure (*Pa*)**	**Oxygen and argon flow rates (mL/min)/(mL/min)**
30	80	0.5	10/80
60			
90			
